# Functional Phenotypes of Intraplaque Macrophages and Their Distinct Roles in Atherosclerosis Development and Atheroinflammation

**DOI:** 10.3390/biomedicines10020452

**Published:** 2022-02-15

**Authors:** Nataliya V. Mushenkova, Nikita G. Nikiforov, Alexandra A. Melnichenko, Vladislav Kalmykov, Nikolay K. Shakhpazyan, Varvara A. Orekhova, Alexander N. Orekhov

**Affiliations:** 1 Unicorn Capital Partners, LLC, 119049 Moscow, Russia; mushenkova@mail.ru; 2 Laboratory of Angiopathology, Institute of General Pathology and Pathophysiology, 125315 Moscow, Russia; nikiforov.mipt@googlemail.com (N.G.N.); zavod@ifarm.ru (A.A.M.); xxor2011@gmail.com (V.K.); 3 Center for Precision Genome Editing and Genetic Technologies for Biomedicine, Institute of Gene Biology, Russian Academy of Sciences, 119334 Moscow, Russia; 4 Laboratory of Clinical Pathology, Institute of Human Morphology, 3 Tsyurupa st., 117418 Moscow, Russia; nshakhpazyan@gmail.com

**Keywords:** atherosclerosis, macrophage, macrophage polarization

## Abstract

Macrophages are the key inflammatory cell type involved in all stages of atherosclerosis development and progression, as demonstrated by numerous studies. Correspondingly, macrophages are currently regarded as a promising therapeutic target for the development of new treatment approaches. The macrophage population is heterogeneous and dynamic, as these cells can switch between a number of distinct functional states with pro- and anti-atherogenic activity in response to various stimuli. An atherosclerotic plaque microenvironment defined by cytokine levels, cell-to-cell interactions, lipid accumulation, hypoxia, neoangiogenesis, and intraplaque haemorrhage may guide local macrophage polarization processes within the lesion. In this review, we discuss known functional phenotypes of intraplaque macrophages and their distinct contribution to ahteroinflammation.

## 1. Introduction

Atherosclerosis is currently recognized as a chronic inflammatory condition, with inflammatory cell types tightly involved in its initiation and progression. The hallmark of chronic atheroinflammation is progressive accumulation of lipids and inflammatory cells in the affected sites of the arterial wall [1]. At the early stages of atherogenesis, plasma low-density lipoprotein (LDL) enters the subendothelial space of the arterial wall, forming lipid deposits. This occurs preferentially at sites of laminar blood flow perturbations, such as bends and bifurcations of the vessel, and/or in the areas of local endothelial dysfunction [1]. Modified LDL, such as oxidized LDL (oxLDL), is especially atherogenic. It is currently assumed that oxidation of accumulated LDL may take place in the arterial wall, with formation of oxidation-specific epitopes which are recognized by the innate immune system, leading to immune activation. These molecules are known as “danger-associated molecular patterns” (DAMPs) [2]. These early events trigger the immune response that involves many cellular subtypes of both innate and adaptive immunity [2]. However, among these various cell types, macrophages and T lymphocytes are the major subsets of inflammatory cells in atherosclerotic lesions [3]. Macrophages were found to be present at all stages of plaque formation, and are currently recognized as a cell type orchestrating the complex process of atheroinflammation.

Macrophages internalize LDL and very low-density lipoprotein (VLDL), as well as oxidized and other modified lipoproteins by means of macropinocytosis, phagocytosis, and scavenger receptor-mediated uptake. This leads to metabolic and functional reprogramming of the cell [3], with decreased phagocytic activity, increased production of pro-inflammatory cytokines, and transformation into foam cells [4]. This type of cell received its name because of the foam-like appearance of the cytoplasm filled with accumulated lipid droplets, and is abundantly present in growing atherosclerotic plaques. In particular, foam cells constitute the primary cell population of fatty streaks, the initial stage in atherosclerosis plaque development. Foam cell formation may be a beneficial adaptive process at the early stages of atherosclerosis, but it appears to be detrimental in advanced lesions due to the massive death of foam cells and release of their pro-inflammatory contents, decreasing plaque stability [5]. Macrophages, as well as dendritic cells, are involved in antigen presentation to T cells, therefore activating the Th1 (T-helper type 1) response and promoting inflammation and atheroma progression. In turn, Th1 cells stimulate pro-inflammatory activation of macrophages by creating a specific cytokine environment [1]. These feedback connections establish a pro-atherogenic signaling cascade responsible for plaque development. Finally, macrophages influence plaque stability by regulating collagen production, the release of matrix degrading enzymes, and induction of smooth muscle cells (SMCs) apoptosis [5]. Even dead macrophages continue to participate in atherosclerosis pathogenesis [6]. At late stages of plaque progression, macrophages contribute to the development of plaque necrotic scores, therefore amplifying the inflammatory response by excessive cell death in the context of defective cell clearance [6].

In an atherosclerotic plaque environment, macrophages are exposed to various signals and stimuli, including cytokines, modified lipids, senescent erythrocytes, and hypoxia, that influence their transcriptional program and functional phenotype. The intensity of these signals changes during plaque progression, and also varies between the plaque regions. As a consequence, intraplaque macrophages undergo polarization in distinct subtypes, often playing opposite roles in atherosclerosis pathology. The concept of macrophage polarization provides a framework for our understanding of the function of macrophages in atherosclerosis development and creates a new paradigm for macrophage targeting for therapeutic purposes.

## 2. Atheroinflammation and Macrophage Polarization Signals

### 2.1. M1/M2 Polarization

Modified lipoproteins within the arterial wall activate resident macrophages and establish a local inflammatory pattern, leading to recruitment of monocytes from the circulation. Plaque macrophage content resembles the balance between blood monocyte recruitment, their differentiation into tissue macrophages, proliferation in situ, retention, migration, and death [7]. Early classification of macrophage polarization defined classically activated, pro-inflammatory M1 and alternatively activated, generally anti-inflammatory M2 phenotypes. Although later studies demonstrated that many more subtypes can be defined based on the differentiation signal, M1/M2 classification can still be used as a rough approximation of functionally opposite states of macrophages [8].

M1 macrophages are induced by Th1 cytokines, such as interferon-γ (IFN-γ), the tumor necrosis factor (TNF), or by lipopolysaccharide or granulocyte macrophage colony-stimulating factors (GM-CSFs) [9]. Data of scRNA-seq analysis have confirmed interleukin (IL)-12–IFN-γ axis is active in human atherosclerotic plaques, driving Th1 activity and M1 polarization of macrophages [10]. Due to the high production of pro-inflammatory cytokines IL-12, TNF, IL-6, and IL-1β, M1 macrophages are important for the establishment of chronic inflammatory states with impaired tissue healing [11]. They are also highly active in the production of reactive oxygen species (ROS) that aggravates intraplaque oxidative stress and tissue damage. Furthermore, M1 macrophages induce the recruitment of Th1 cells due to the expression of the chemokine (C-X-C motif) ligand (CXCL)9, CXCL10, and CXCL5 [12]. M1 macrophages have decreased migration activity, and their accumulation and death contribute to necrotic core formation in the plaque interior, which is a key feature of progressing plaques [12]. Advanced glycation end products (AGEs) were also described as a M1 polarizing signal [13]. AGEs are irreversible products of the nonenzymatic glycation and oxidation of proteins, lipids and nucleic acids that activate receptor-of-AGE (RAGE) signaling. The AGE/RAGE axis may be important for atherosclerosis development in patients with diabetes mellitus [13].

Pro-inflammatory and tissue-damaging activity of M1 macrophages is counterbalanced by the so-called alternatively activated, or M2 macrophages. Alternative M2 macrophages differentiate in the presence of Th2 cytokines, such as IL-4 or IL-13. They dampen the inflammatory Th1 response by producing anti-inflammatory factors IL-10, transforming growth factor (TGF)-β, IL-1 receptor antagonist (IL-1Ra), and promote angiogenesis and tissue repair. LDL receptor-related protein (LRP)5, a member of the LDL receptor superfamily, and a Wnt co-receptor was shown to be a marker of the M2 subtype in human atherosclerotic plaques [14]. LRP5 expression is found in advanced human atherosclerotic lesions, co-localizing only with the CD16^+^ macrophage subset [14]. LRP5^+^ cells infiltrate deep layers of the plaque and possess high phagocytic activity. This marker may also be relevant for murine M2 populations, as anti-inflammatory CD115^+^GR1^low^ monocytes in mice under normal conditions exhibit increased LRP5 expression compared to pro-inflammatory CD115^+^GR1^high^ cells. When fed on a hypercholesterolaemic diet, LRP5-deficient (*Lrp5^−/−^*^)^ mice develop larger aortic lipid accumulation than wild-type animals, along with downregulated Wnt/β-catenin signaling, higher macrophage numbers in the aorta, and enhanced production of pro-inflammatory mediators by blood monocytes [15]. In vitro inhibition of the Wnt/β-catenin pathway increases the production of pro-inflammatory cytokines in monocytes and peripheral blood mononuclear cells [15], suggesting Wnt signaling to be one of the macrophage polarizing pathways.

**Figure 1:**
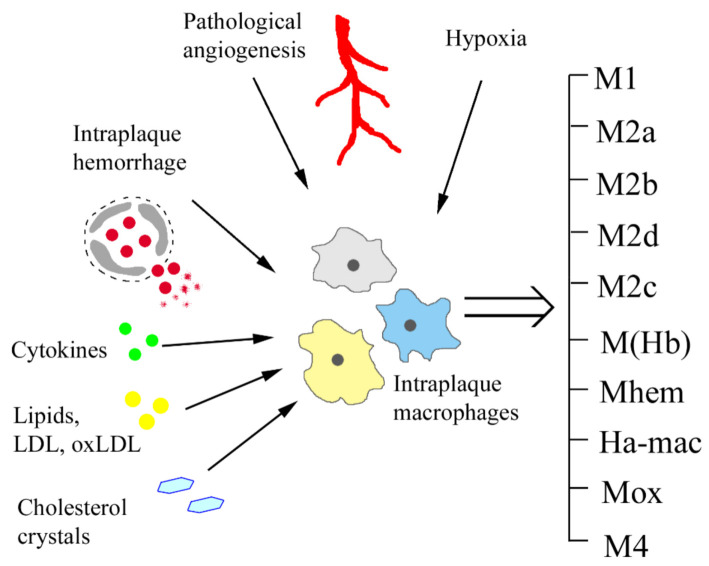
The variety of intraplaque macrophage phenotypes develops in response to different environmental stimuli. LDL, low-density lipoprotein.

Although M1/M2 dichotomy was useful to describe two opposite pro- and anti-inflammatory states of macrophages identified in vitro [9], the in vivo situation appears to be more complex, with a continuum of macrophage phenotypes present in the sites of inflammation that develop in response to various stimuli (Figure 1). Moreover, macrophages retain plasticity and can switch between different functional phenotypes according to local cytokine milieu. Classification of macrophages is not an easy task, as majority of markers are not exclusive for one phenotype and M1 and M2 signatures do not necessarily exclude each other and often coexist [8]. At present, macrophage phenotypes are usually characterized on the basis of marker expression combined with functional studies. Furthermore, surface markers may differ between species, though the general properties of macrophage subpopulations are conserved (Table 1).

Type M2 macrophages can be further subdivided into four different subclasses: M2a, M2b, M2c, and M2d. M2a are induced by IL-4 and IL-13 and are characterized by a high level of macrophage mannose receptor (MMR) expression. They secrete pro-fibrotic factors, such as fibronectin, insulin-like growth factor, and TGF-β, which are important for tissue repair and are classically recognized as “wound healing macrophages” [8]. IL-4, a potent inducer of the M2a phenotype, was shown to be expressed in human atherosclerotic plaques [11]. M2b macrophages are induced by immune complexes in combination with IL-1β or toll-like receptor (TLR) ligands. M2b are unique among M2 subtypes as, in addition to IL-10, they also produce higher levels of pro-inflammatory cytokines, including IL-1β, IL-6, and tumor necrosis factor (TNF). M2b-dependent IL-10 production was shown to be important for atheroprotective responses in mice [16]. M2c macrophages differentiate in the presence of IL-10, TGF-β, or glucocorticoids [11]. Expression of Mer tyrosine kinase (MerTK), a major macrophage apoptotic cell receptor, was shown to be restricted to this population. MerTK enables M2c macrophages to clear apoptotic cells more efficiently than other macrophage subsets, thus resembling an important checkpoint in the maintenance of an anti-inflammatory environment [17]. While M2a, M2b, and M2c macrophages were found in both humans and mice, the M2d phenotype was identified in mice only. M2d is induced in the presence of the adenosine A2 receptor and TLR agonists, and is not driven by IL-4 [18,19]. Adenosine signaling suppresses TLR-dependent expression of TNF, IL-12, IFN-γ, and several other inflammatory cytokines, and induces expression of the vascular endothelial growth factor (VEGF), IL-10, and inducible nitric oxide synthase (iNOS), therefore endowing M2d with pro-angiogenic activity [18].

At present, the specific roles of different M2 subpopulations in the context of atheroinflammation are not defined. All M2 macrophage phenotypes are characterized by a low level of IL-12 and high production of both IL-10 and TGF-β that are anti-inflammatory. M2 plays a major role in the elimination of apoptotic cells and cell debris, possesses pro-fibrotic function, and promotes tissue healing. However, M2 polarization is not purely atheroprotective, as these cells can actively produce pro-angiogenic factors and participate in matrix remodeling, thus promoting plaque growth and inflammation [20].

Spatial distribution within the atherosclerotic plaque can be different for different macrophage phenotypes [21], with M1 being predominantly found in the lesion shoulder, one of the most unstable areas within the plaque. Both M1 and M2 markers were shown to be present in the fibrous cap surrounding the necrotic core, indicating that a balance exists between pro-inflammatory and profibrotic plaque-stabilizing effects [22]. In the adventitia, M2 macrophages are two- to threefold more abundant than M1 macrophages [22]. Studies in mice have suggested that this difference in macrophage subtypes distribution can result from an in situ phenotype switch depending on the local environment, and not the difference in the migration patterns of macrophage subtypes [23]. This is also supported by the study of long-term macrophage positional dynamics within the plaque by tracking the displacement of nondegradable phagocytic particles within macrophages with intravital imaging [24]. The results showed that newly recruited monocytes were stopped within the superficial layers failing to penetrate significantly deeper, and intraplaque macrophages remained for a long time in the same area. Waves of recruited monocytes create layers reminiscent of growth rings in trees, and may have no contribution to the processes taking place in deeper layers. Therefore, phenotype segregation between the plaque regions, with classically activated macrophages found in unstable regions, such as the necrotic core, and alternatively activated macrophages near the stable regions, is a result of stable localization in the areas with different microenvironments [24].

Though macrophages are traditionally classified into five subtypes (M1, M2a, M2b, M2c, and M2d), more phenotypes were described that are relevant for the pathogenesis of atherosclerosis (Table 1). Macrophage polarizing signals specific for atheroinflammation are discussed below.

### 2.2. Lipids as Macrophage Polarizing Signal

The extravasation of oxLDL into the arterial wall is a key event in the pathogenesis of atherosclerosis that begins prior to the formation of visible plaque lesions and continues throughout the entire course of plaque development. Exposure to accumulating lipids and their oxidized derivatives is one of the driving stimuli of intraplaque macrophages polarization. Modified LDL can be sensed by macrophages through different scavenger receptors, including scavenger receptor A1 (SRA1), scavenger receptor B1 (SRB1), CD36, and others, all of which can promote foam cell formation [12]. Responses to native and modified lipids may vary depending on the lipid derivative, but mostly, they drive M1 polarization by different mechanisms [11]. Cholesterol crystals can trigger activation of NLR family pyrin domain containing 3 (NLRP3) inflammasome, resulting in the release of IL-1 family cytokines [12]. Intracellular accumulation of cholesterol in the context of diminished cholesterol efflux also leads to the development of endoplasmic reticulum (ER) stress and cell death. Increased apoptosis of macrophages caused by cholesterol-induced ER stress could be atheroprotective at early atherosclerosis stages in the case of effective efferocytosis of dead cells. However, in advanced atherosclerotic lesions, the combination of lipid toxicity and defective cell clearance is critical for necrotic core formation and growth, which contributes to advanced unstable plaque formation. Cholesteryl esters (including linoleate and 7-ketocholesteryl-9-carboxynonanoate) induce M1 polarization by activating the TLR-4 and nuclear factor (NF)-κB signaling pathways [11]. Accumulation of oxLDL also directs macrophage polarization towards M1 phenotype, the mechanism was shown to be mediated by inhibition of the transcription factor Kruppel-like factor 2 [11]. Conversely, a number of lipids and their derivatives may be involved in the M2 switch. This activity was described for 9-oxononanoyl-cholesterol, a major cholesteryl ester oxidation product [12], resolvin D1, ω3-polyunsaturated fatty acid derivative, sphingosine-1-phosphate (S1P), and conjugated linoleic acid [11].

Liver X receptors (LXRs) represent an important link between cholesterol accumulation and inflammatory response in macrophages. LXRs sense cholesterol derivatives, such as oxysterols and desmosterol. Due to upregulation of genes driving cholesterol efflux and anti-inflammatory pathways, LXRs are thought to be atheroprotective in mice. In humans, the situation appears to be more complex. LXR agonist treatment induced IL-1β expression in M1 as well as M2 polarized macrophages, and this effect was shown to be dependent on the hypoxia-inducible factor 1α (HIF-1α) [25]. LXR was shown both to upregulate HIF-1α expression and to stabilize HIF-1α by direct interaction with the oxygen-dependent degradation domain of HIF-1α. Macrophage exposure to a LXR agonist induced a broad transcriptional response with upregulation of several pathways connected with glycolysis and angiogenesis, known to be HIF1-regulated. These results indicate a possible proatherogenic role of LXR in macrophages that is human-specific [25].

In addition to M1 and M2 phenotypes, macrophages exposed to oxidized phospholipids may be switched to the Mox phenotype, characterized by reduced phagocytic activity and chemotaxis [11]. Mox differentiation is driven by transcription factor NFE2L2 [11]. Transcriptomic analysis has shown that out of 119 upregulated genes during Mox differentiation, only 38 overlap between M1 and Mox, and 13 are similar between Mox and M2 [26]. Genes exclusively activated in Mox macrophages include a set of redox-regulated genes, such as heme oxygenase 1 (*Hmox-1*), thioredoxin reductase 1 (*Txnrd1*), sulfiredoxin (*Sxrn-1*), regulatory and catalytic subunits of glutamate-cysteine ligase, and others, such as nuclear receptor 4A2 (*Nr4A2*), *Vegfa* and Tribbles homolog 3 (*Trib3*). Mox have both pro-inflammatory (COX-2, IL-1β production) and atheroprotective activities (regulation of intraplaque redox status). In low-density lipoprotein receptor (*Ldlr*)-deficient mice, this population constitutes up to 30% of macrophages present in advanced atherosclerotic lesions [26], with the M1 phenotype accounting for 40%, and M2 for 20%. The relevance of Mox macrophages for human pathology is unknown.

Oxidized lipids may decrease phagocytic activity of macrophages not only through their polarization to the Mox phenotype, but also directly by competitive inhibition of receptors involved in apoptotic cell clearance, or efferocytosis, which is one of the basic functions of macrophages [9]. Efferocytosis in early lesions is usually efficient, leads to elimination of dead macrophages before they become secondary necrotic, and is accompanied by an anti-inflammatory response. However, in advanced lesions, phagocytic clearance is not efficient, leading to accumulation of necrotic cells and plaque progression [9]. Stimulation of efferocytosis may be an effective therapeutic strategy, as was shown in the study by Flores et al. [27]. Nanoparticles with the SH2 domain-containing phosphatase-1 (SHP1) inhibitor were used to target the CD47-SIRPa (signal regulatory protein-α) pathway of phagocytosis regulation. CD47 functions as a ligand for SIRPα expressed on macrophages, which signals through SHP1 phosphatase to suppress phagocytosis [28]. Nanoparticles were shown to accumulate in plaque resident phagocytes, resulting in significant anti-atherosclerotic effects in two independent murine models of atheroinflammation [27]. Lesions from treated mice displayed three-fold smaller necrotic cores and two-fold reduced accumulation of apoptotic cells. Decreased arterial inflammation was confirmed by in vivo 18F-fluorodeoxyglucose positron emission tomography/computed tomography imaging. Single-cell RNA sequencing revealed that chronic efferocytosis stimulation elicited numerous changes in lesion-resident macrophages, including a decrease of pro-inflammatory transcripts (*Ccl2, Ccl7, Ccl8, Pf4*), genes of IL-1β and INF-γ response and upregulation of genes linked to inflammation resolution (*Socs3, Zfp36*) and phagocytosis [27]. The results of this study provided an important indication that modulation of macrophage functional pathways may be effective for changing the evolution of the plaque.

### 2.3. Hypoxia as Macrophage Polarizing Signal

Hypoxia develops from the early stages of plaque formation as a result of intimal thickening [29]. In response to tissue hypoxia, angiogenesis involving the vasa vasorum vasculature is activated. However, new blood vessels formation is not sufficient to support adequate oxygen supply throughout the plaque, as it is limited to the base and shoulder areas of a lesion, leaving the core avascular and triggering massive necrotic cell death [29]. Hypoxia areas were shown to colocalize with CD163, a specific macrophage marker, and was prominently higher in atherosclerotic lesions compared to normal vessels [29]. Due to positive feedback loop between plaque growth and hypoxia stress, atherosclerosis appears to be a self-perpetuating pathological process. Hypoxia-induced vascularization by vasa vasorum vasculature provides routes for circulating monocytes to infiltrate into the plaque. Analysis of early atherosclerotic plaques of human carotid arteries supports this mechanism. Capillary-like microvessels were shown in very early atherosclerotic lesions (type II). In both type II and III lesions, macrophages and foam cells accumulated in perivascular areas [30].

HIF-1α is a master regulator of hypoxic response genes. As previously described, hypoxia is not a sole factor responsible for HIF1α activation in plaque macrophages. In fact, HIF-1α immunoreactivity and mRNA were detected at sites of inflammation even in the areas close (20–30 μm) to the vessel lumen, well below the oxygen diffusion limits (100–250 μm) [31]. HIF-1α promotes atheroinflammation by different mechanisms. HIF-1α is responsible for induction of pro-inflammatory cytokines that mediate the recruitment of monocytes into the plaque, such as stromal cell-derived factor 1 (SDF-1), TNF, IL-1β, VEGF, and monocyte chemoattractant protein 1 (MCP-1), as well as induction of macrophage retention molecules, including netrin-1, Unc5b, and semaphorin 3E. In vitro hypoxia dramatically promoted the adhesion of human monocytes to the endothelial cells, as well as chemotaxis, through a mechanism dependent on the upregulation of RAGE and NF-kB activation [32]. HIF-1α reduces mRNA expression of the major cholesterol transporters and activates the lectin-like oxLDL receptor-1 (Lox-1) that mediates oxLDL uptake in macrophages [33]. Under normal conditions, macrophages are able to esterify free cholesterol into cholesteryl esters for storage in the form of lipid droplets. However, cholesteryl ester levels were found to be significantly lower in hypoxic macrophages with a higher percentage of unesterified cholesterol. HIF-1α upregulate transcription of hypoxia-inducible protein 2 (HIG2)/hypoxia-inducible lipid droplet-associated (HILPDA), a protein localized to the endoplasmic reticulum–lipid droplet (LD) interface, the site of LD formation [34]. HILPDA was shown to be crucial to foam cell differentiation and lipid deposition. Proatherogenic role of HILPDA was confirmed in vivo in apolipoprotein E (ApoE)-deficient mice [34].

HIF-1α was shown to be associated with the production of proteoglycans by macrophages, such as versican, that are important for retention of atheropathogenic apoB-100-containing lipoproteins in the arterial wall [35]. In addition, hypoxia altered the modification of glycosaminoglycan favoring the formation of more negatively charged matrix that was more sulphated and displayed a higher affinity to LDL [35].

In vitro data clearly support the idea that hypoxia stimulates M1 polarization [32], enhancing expression of M1 markers. IL-6 mRNA expression was increased by approximately 3.2-fold, while TNF-α, IL-1β, and CD80 by around 40%, 75%, and 120%, respectively, versus normoxia conditions [32]. At the same time, no significant change in the expression of M2 macrophage markers was observed [32]. HIF-1α deficient cells showed a lower expression of M1-typical genes compared to control cells, with no difference in M2 polarization [35].

A mechanism connecting HIF-1α activation in lesion macrophages and necrotic core formation was described recently [36]. MicroRNA expression profiling showed that HIF-1α upregulated miR-210, that is known to inhibit oxidative phosphorylation and enhance mitochondrial ROS production. The target of miR-210 is 2,4-dienoyl-CoA reductase, an enzyme participating in β oxidation of fatty acids. Critical reduction in mitochondrial energy production potentiated Rip-3 (receptor-interacting serine/threonine-protein kinase 3)-mediated necroptosis of macrophages [36].

Activation of HIF signaling can also induce the endothelial-to-mesenchymal transition (EMT) of endothelial cells, defined by the acquisition of stem cell (Sca1, Snail1) and mesenchymal (smooth muscle actin, SMA) markers, collagen and fibronectin production and fibroblast-like morphological changes. EMT characteristics have been discovered in human atherosclerotic lesions [10]. In mice, EMT was shown to affect macrophage functions [37], though, at present, the available experimental data are scarce and unconclusive. EMT has a different pattern of polarizing cytokine production in comparison with endothelial cells, with downregulation of M-CSF (classical M2 polarizing factor), increase in GM-CSF (classical M1 polarizing factor) and TGF-β, IL-10 (M2 -associated cytokines) [37]. Decreased TNF production in response to oxLDL reduced the expression of antigen presenting cell markers and increased efferocytosis in response to factors secreted by EMT cells, which may promote a less inflammatory phenotype. At the same time, enhanced macrophage lipid uptake and accumulation may be connected with a pro-inflammatory switch [37].

In vivo data confirmed that hypoxia can be an important factor of plaque growth and instability. In *apoE^−/−^* mice maintained on high cholesterol diet, induced hypoxia (10.0% O_2_) accelerated plaque growth [38]. Targeting of HIF by FG-4497 inhibitor (developed by Fibrogen) in LDL receptor-deficient mice led to a 50% reduction of atherosclerotic plaques area versus vehicle-treated mice. A therapeutic effect was correlated with the reduction in weight, serum cholesterol levels, and white adipose tissue macrophage aggregates and increase in protective autoantibodies [39]. Transient local inhibition of HIF by using a dominant-negative mutant at the early stage of atherosclerosis dramatically reduced the extent of neointima formation in *apoE^−/−^* mice with vascular injury. In contrast, overexpression of HIF-1α and HIF-2α was accompanied by increased plaque growth [40].

Therefore, hypoxia is a pathogenic factor in atherosclerosis lesion development. There is a clear correlation between the growth and potential rupture of the atherosclerotic plaque and the expression of HIF-1α, a central regulator of the hypoxic response. HIF-1α activation in plaque macrophages induced by hypoxia or other factors is connected with a metabolic switch towards glycolysis and pro-inflammatory polarization. An important consequence of hypoxia and HIF-1α stabilization is upregulation of VEGF, a central cytokine involved in neoangiogenesis. VEGF was shown to be a key factor of vascular fragility and permeability [31], which are atherosclerosis features associated with intraplaque haemorrhage.

### 2.4. Haemorrhage as Macrophage Polarizing Signal

Intraplaque haemorrhage (IPH) is usually present in atherosclerotic lesions as a consequence of active neovascularization with the formation of abnormal leaky vessels prone to rupture [41]. IPH is a feature of advanced, rapidly progressing plaques [42]. Iron overload strongly promotes the shift toward the M1 phenotype [43]. Iron was described as an M1-polarization factor in a number of pathological states [43,44,45], including atherosclerosis [46]. A correlation between the level of inflammatory macrophages infiltration, plaque iron and more advanced plaque state was observed in patients with carotid atheroma [47]. M1 macrophages maintain high intracellular iron levels, and, in response to iron overload, increase the expression of scavenger receptors, leading to foam cell formation. Iron accumulation by M1 leads to enhanced production of ROS and potentiation of tissue damage. It was also shown that atheroinflammation could increase the local production of hepcidin, a key regulator of intracellular iron retention. This potentiates NF-kB signaling and inflammatory cytokines release by M1 macrophages. The hepcidin-mediated accumulation of iron in plaque macrophages and the resulting inflammation could constitute a self-amplifying process that promotes atherosclerosis [48].

M2 macrophages are able to metabolize haem iron via heme oxygenase-1 (HO-1). Due to HO-1-mediated degradation, haem is transformed in biliverdin and subsequently converted into bilirubin, which has anti-inflammatory and anti-oxidant properties [48]. Free iron is either sequestered via ferritin or exported via the ferroportin transporter (FPN) leading lo low-cellular-iron concentrations [31].

Within IPH areas, free hemoglobin (Hb) released as a result of erythrocyte lysis forms a complex with the plasma protein haptoglobin (Hp), and haemoglobin:haptoglobin complexes are internalized by macrophages via scavenger receptor cysteine-rich type 1 protein M130 (CD163). Intake of haemoglobin:haptoglobin complexes or haem by macrophages is a polarization signal resulting in formation of distinct macrophage phenotypes HA-mac, M(Hb) and Mhem (Table 1).

M(Hb) macrophages express both macrophage mannose receptor 1 (MMR, CD206) and CD163 [49]. M(Hb) subtype is characterized by the production both anti-inflammatory (IL-10, IL-1Ra) and pro-inflammatory (VEGF, IL-1β) factors [9]. These cells produce less ROS than other macrophage subtypes and have low iron accumulation because of the upregulation of FPN [49]. Due to increased activity of LXR-α and induction of cholesterol efflux, these macrophages are protected against lipid accumulation. M(Hb) accumulate in the areas of intraplaque angiogenesis and increased vessel permeability and were shown to be proangiogenic.

Haem directs macrophage polarization towards the Mhem phenotype [50]. This switch is driven by the induction of activating cyclic AMP-dependent transcription factor ATF-1 and oxysterols receptor LXR-β. Mhem macrophages are characterized by increased expression of atheroprotective enzyme HO-1. Similar to M(Hb) subtype, Mhem macrophages express CD163 and are protected from iron and lipid accumulation. Some authors did not distinguish Mhem and M(Hb) populations [20].

HA-mac was described as CD163^high^HLA-DR^low^ population with athroprotective phenotype [51]. Differentiation of HA-mac is driven by threshold levels of haemoglobin:haptoglobin and depends on IL-10/CD163-positive feedback loop [52].

HA-mac, M(Hb) and Mhem macrophages phagocytize and utilize erythrocyte remnants and haemoglobin deposits. These macrophages were initially thought to be atheroprotective due to IL-10 release and low production of pro-inflammatory cytokines. This concept was challenged by the study of Guo et al. [20], showing that Hb-activated CD163^+^ macrophages promote angiogenesis and vascular permeability. CD163^+^ macrophages probably contribute to plaque instability through a pathway involving iron-mediated HIF-1α protein stabilization and subsequently increased VEGF production [53]. VEGF acting through VEGF receptor (VEGFR)2 provokes a loss of endothelial barrier function and inflammatory cell recruitment into the vascular wall. Analysis of human atherosclerotic samples showed a strong association between the CD163^+^ macrophage area and features of plaque progression, such as the necrotic core, presence of ruptures, or pathological intimal thickening [20]. In accordance with the proposed pathogenic mechanism, the microvessel number was significantly greater within CD163-positive areas. The finding of RS7136716 GG polymorphism of CD163, which increases CD163 expression, being an independent risk factor of coronary plaque rupture and plaque angiogenesis, also supports the suggestion about deleterious role of CD163^+^ macrophages in atheroslcerosis. A direct experimental proof was achieved in *apoE^−/−^* mice crossed with CD163^−/−^ mice. Atherosclerosis development was monitored in brachiocephalic arteries (BCA). In comparison with *apoE^−/−^* mice, CD163^–/–^ *apoE^−/−^* double knock-out mice had more than 2-fold reduction in plaque lesion area and nearly 3-fold decrease in pathological score, accompanied by reduced intraplaque neovascularization and haemorrhage [20].

Sites of neovascularization are also characterized by a high level of CXCL4, which is abundantly expressed in macrophages and the neovascular endothelium. In ApoE-deficient mice, deletion of CXCL4 results in a decrease in plaque size, indicating the proatherogenic effects of this chemokine. In humans, CXCL4 induces the M4 macrophage phenotype that shares some characteristics with M1 and M2, but does not possess phagocytotic activity. The transcriptome of M4 macrophages is not clearly pro- or anti-atherogenic, but is distinct from that of M1 and M2 subsets [54]. M4 macrophages might prevent the development of macrophages of the Mhem phenotype and have pro-inflammatory activity, releasing IL-6 and TNF-α. M4 macrophages express low levels of scavenger receptors essential for the uptake of modified lipids, but high levels of the cholesterol efflux transporter ABCG1, suggesting they are resistant to foam cell formation. In contrast to the labile switch between M1/M2/Mox subtypes, polarization to M4 was shown to be irreversible [54]. Macrophage CXCL4 expression positively correlates with clinical parameters such as lesion grade and the presence of symptomatic atherosclerotic disease in human carotid atherosclerotic plaques [54].

Red blood cell (RBC) membranes have a high cholesterol content with a percentage of lipids up to 40% of the total weight of the cells [41]. RBC membranes may be important contributors to lipid deposition and lipid core expansion upon IPH. This suggestion is supported by the data on colocalization of cholesterol crystals within the plaques with iron and glycophorin A, a characteristic protein of the RBC membrane [41].

IPH may be targeted by anti-angiogenic treatment. This strategy was rather powerful in animal models of atherosclerosis [55,56,57]. For instance, VEGFR2 blockade was studied in the model of hypercholesterolaemic ApoE3*Leiden mice that received a donor caval vein interposition in the carotid artery. VEGFR2 blockade in this model resulted in a significant 44% decrease in IPH, accompanied by 32% reduction in vein graft size, development of more stable lesions with reduced macrophage accumulation and higher collagen content [55]. More research is needed to understand the perspectives of anti-angiogenic treatment and develop most effective and safe strategies of neoangiogenesis targeting [58].

## 3. Single-Cell Level of the Diversity of Intraplaque Macrophages

The discovery and rapid progress of single-cell RNA sequencing (scRNAseq) and tools exploiting large combinations of protein labelling of cells, such as cytometry by time of flight (CyTOF), have now allowed classification of macrophages based on transcriptional patterns or protein marker sets and thus provided a more detailed description of intraplaque macrophage phenotype and functional diversity.

Studies of atherosclerosis development in *Ldlr^−/−^* or *ApoE^−/−^* mice with CyTOF or scRNAseq approaches confirmed previous histological findings, showing that macrophages are the most prevalent immune cell type within the atherosclerotic plaque [59,60] and the total proportion of macrophages increases with the evolution to more advanced stage [60]. Myeloid cells, including macrophages and monocytes, were underrepresented in digestion-based scRNAseq and flow cytometry studies, with CD11b^+^ cells comprising 20% of plaque leukocytes. At the same time, the genetic deconvolution method gave a more representative picture of plaque composition, showing the frequency of myeloid cells of more than 75% [61]. Summarizing currently available single-cell data from murine atherosclerosis models, three main subtypes of intraplaque macrophage could be identified, including resident-like macrophages, pro-inflammatory and foamy triggering receptor expressed on myeloid cells (TREM)2hi macrophages (Table 2). Further subdivision of these three main types is possible [59].

Resident arterial macrophages emerge during embryonic development. They retain expression of the precursor marker CX3C chemokine receptor (CX3CR)1 and specifically express Lymphatic vessel endothelial hyaluronan receptor 1 (Lyve1). Depending on the study, additional markers of this population were identified: mannose receptor MMR (Cd206), transcription factor Mafb [62], Factor XIIIa (F13a1), Growth arrest-specific 6 (Gas6) [60], and others. According to their tissue-resident subtype, these macrophages are identified both in normal and atherosclerotic areas of the aorta [60], and they are predominately present in the adventitia. It is not clear whether migrating monocytes may acquire resident-like phenotype. Increased expression of Ccr2, a marker for recruited macrophages, may point to this possibility. Several studies described that resident-like macrophages can proliferate by showing either protein expression of proliferation marker Ki-67 or enrichment for cell cycle genes [59]. Folr2, Cbr2, Sepp1 and Cd206 expressed by lesion resident-like cells are all associated with M2-like phenotype, suggesting anti-inflammatory characteristics of the population. There was no difference in the proportion of resident-like macrophages between progressive and regressive plaques [59].

Different single-cell studies confirmed the presence of inflammatory macrophages in atherosclerotic lesions [59] and were concordant in the description of this subset. It is enriched with transcripts of classical pro-inflammatory pathways (Il1α, Il1β, Tlr2, Tnf), chemokines (Ccl2–5, Cxcl1, Cxcl2, Cxcl10) and interferon I signaling genes. The relative frequency of the inflammatory macrophage subset positively correlates with plaque progression [59]. A rather unexpected finding was the absence of association of inflammatory patterns with foam cell phenotype (discussed below). In contrast to resident-like population, the inflammatory population is absent in normal vessels, being present only in atherosclerotic lesions, where they represent the largest macrophage subset [60]. They also differ in the localization, with predominant presence in the intima, including plaque shoulder regions [59]. According to the transcriptional and functional profile, this population closely resemble classical M1 phenotype.

TREM2hi macrophages were identified exclusively in the plaques and not in the healthy aorta [60]. TREM2 is a myeloid-specific transmembrane glycoprotein, that works as a lipid sensor, binding apolipoprotein E, glycerophospholipids, sphingomyelins. TREM2 was described as a marker of lipid-associated macrophages differentiated in obesity, driving gene expression program involved in phagocytosis, lipid catabolism and anti-inflammatory phenotype [63]. According to current understanding, TREM2 pathway may represent a conserved macrophage response for detection of extracellular pathogenic lipids across multiple tissues [63]. Pathway analysis of TREM2hi lesion macrophages showed connections with foam cell phenotype and enrichment in transcripts involved in cholesterol metabolism and oxidative phosphorylation [59]. Foam cell characteristics of TREM2hi macrophages were confirmed by independent studies [60,62]. This subset of cells resides in the intima, being involved in the uptake of atherogenic lipoproteins and lipid-rich core formation. Comparison of transcriptomic profiles of intimal foamy and non-foamy macrophages sorted from pooled atherosclerotic aortas of *ApoE^−/−^* mice showed that inflammatory genes such as *I1b*, *Nfkbia*, *Tlr2*, and *Tnf* were mostly upregulated in non-foamy population, while foam cells were enriched in resolving/regression-related genes [62]. The notice that foam cell formation is not a pro-inflammatory process is a recent ongoing paradigm shift in the atherosclerosis field, as several studies have previously shown clear pro-inflammatory characteristics of foam cell formation, which was, for a long time, regarded as a potential target for atherosclerosis treatment [64]. Although foamy macrophages are anti-inflammatory with M2-like features, their accumulation positively correlates with the severity of atherosclerosis [62].

Macrophage subsets defined by Fernandez et al. [65] in human carotid endarterectomy specimens are close to murine classification discussed above. In contrast to murine models, in human plaques, macrophages were not the major population among CD45^+^ cells, comprising less than 20% [10,65]. The study described the same three populations of intraplaque macrophages [65]. The CD206^hi^CD163^hi^ macrophage subset may be similar to resident-like macrophage subset based on CD206 expression and absence of foam- cell patterns. The pro-inflammatory macrophages in human plaques expressed increased levels of activation markers, such as major histocompatibility complex class II DR alpha (HLA-DRA), CD74, cytochrome B-245 alpha chain (CYBA), lysozyme C-2 precursor (LYZ2), allograft inflammatory factor (AIF)1, S100A8/A9, metastasis associated lung adenocarcinoma transcript (MALAT)1, JUNB, NFKBIA. The foamy anti-inflammatory macrophage subset was enriched for the expression of LGALS3 (galectin 3 gene) and foam cell-related transcripts such as apolipoprotein C (APOC)1, APOE, cathepsin B (CTSB), FABP5 and perilipin 2 (PLIN2). TREM2 and CD9 expression in foamy subset was observed in another study, closely corresponding to the data from murine studies. Characteristics of foamy-like population also included the upregulation of metabolic pathways and liver X receptor/retinoid X receptor (LXR/RXR) activation, as well as STAT6-driven anti-inflammatory pathways [10].

Macrophage transcriptional profiles may differ between patients with asymptomatic lesions (ASYM) and those having cerebrovascular complications (SYM) [65]. ASYM macrophages were more activated, pro-inflammatory with high IL-1β signaling (IL-1β, IL-1RAP, NLRP3 expression) and displayed enhanced foam cell functions compared to SYM macrophages. In SYM plaques, macrophages expressed several genes associated with plaque instability: granzymes, CCL5, pro-angiogenic factors IL-8 and CXCR2, genes involved in Hedgehog and Wnt signaling. T cell-macrophage and macrophage- macrophage cell-to-cell communications were described that may be associated with ASYM and SYM plaques [65].

Single-cell RNA-Seq was combined with genetic fate mapping of myeloid cells derived from CX3CR1^+^ precursor monocytes to analyze the differences in transcriptional programs between progressing and regressing plaques in mice. Bone marrow chimeras of *ldlr^–/–^* mice reconstituted with bone marrow from Cx3cr1CreERT2-IRES-YFP/+Rosa26fl-tdTomato/+ mice were used that allow to map all CX3CR1+ macrophages with tdTomato expression upon tamoxifen exposure. No significant difference in the overall number TdTomato+ cells from aortas of mice undergoing progression and regression was found, but regressing lesions were characterized by increased number of macrophages with M2 markers (PD-L2, CD301) [66]. The cluster that resembled resident-like macrophages (Folr2hi macrophages) was the highest in both progressing and regressing lesions. There was a considerable heterogeneity of macrophage activation states under progression conditions possibly reflecting more complex polarizing environment. An interesting finding was the identification of a specific M2 subpopulation (Retnla^hi^Ear2^hi^ macrophages) in progressing plaques that was actually absent from regressing plaques, that challenged the idea of plaque progression as solely M1-driven process. A specific cluster of M2 polarized cells associated with regressive plaques was also identified, that expressed stabilin-1 (Stab1) and selenoprotein-1 (Sepp1), proteins with efferocytosis-enhancing and anti-inflammatory activities. A cluster of proliferating CX3CR1^+^ cells with stem cell–like signature expressing markers of both monocytes and macrophages was described. This fact broadens our view of the origin of proliferating macrophages within the plaque [66].

## 4. Future Perspectives

Given the central role of macrophage phenotypes in the development of atherosclerotic plaques and in processes leading to plaque instability and rupture, targeting macrophages for development of novel therapies is an attractive field of future research. A major challenge to such research is the diversity of macrophage population. Animal models commonly used to study atherosclerosis, such as mice, do not accurately reproduced the macrophage subsets observed in humans [67]. The use of modern genetic engineering techniques allowed creation of more specific models that, although no reproducing the complexity of human situation, can be used for studying certain aspects of atherosclerotic process, such as plaque instability and rupture, and the involvement of macrophages in these processes [68]. Detailed study of macrophage subsets in such models may provide information for more targeted approach in therapy development.

Another important direction of future research is better characterization of macrophage phenotypes and their functions, since many aspects of this complexity remain poorly understood. Pro-inflammatory M1 macrophages can have inconsistent effects on tissue healing and regeneration [69,70]. Alternatively-activated M2 macrophages can have beneficiary effects through tissue regeneration, but are also known to be implicated in fibrosis [71]. Macrophage involvement in the various aspects of plaque development, such as inflammation, phagocytic intake of lipids, foam cell formation, fibrogenesis, regeneration and tissue remodeling should become the focus of future studies.

The effect of available therapies on macrophage polarization is being actively studied. Macrophage reprogramming towards the anti-inflammatory (M2) phenotype in particular is considered as a possible therapeutic strategy. This approach achieved considerable effects on plaque reduction in *ldlr^−/−^* mice, but needs to be validated in humans [72]. Macrophage reprogramming, cholesterol efflux stimulation and inflammation inhibition can also be achieved by treatment with HDL, as demonstrated in murine models [73,74]. The effects of HDL on macrophage plaque population in humans is the subject of ongoing clinical trials. Since statins are known to have pleiotropic effect with pronounced anti-inflammatory properties, numerous studies have evaluated their relation to plaque macrophages. However, a recent review argued that the plaque regression induced by statins is probably due to the indirect, lipid-lowering effects rather than to direct action on plaque macrophages [75]. At the same time, using statins in a more targeted way may still prove to be beneficial. A recent study with atorvastatin-loaded modified HDL particles resulted in an anti-inflammatory, cholesterol efflux-increasing effect in cultured macrophages, although confirmation in relevant models remains to be obtained [76]. Another in vitro study demonstrated that different statins (pitavastatin, atorvastatin, fluvastatin, and lovastatin) could inhibit the uptake of carbon nanoparticles and cholesterol crystals by macrophages. This led to reduced production of IL-1β therefore providing for another possible anti-inflammatory effect of statins [77]. These results indicate that the pleiotropic actions of statins are worthy to be studied further and may manifest especially at a cellular level, specifically affecting plaque macrophages.

Another direction of future studies in enhancing the effect and selectivity of anti-inflammatory therapies is to target vascular inflammation through macrophage polarization, for instance, involving nanoparticle delivery technology. A recent study demonstrated that co-delivery of baicalein (an anti-inflammatory drug) with anti-miR155 in a form of nanocrystals showed a potent effect on anti-inflammatory macrophage polarization in vitro and in vivo [78]. Anti-inflammatory effects of platelets can also be explored in a relationship to macrophage polarization. It was shown that platelet-conditioned media (with cultured healthy human platelets) induced anti-inflammatory pro-resolving macrophage phenotype [79]. More translational and clinical studies are needed to explore these interesting opportunities and, possibly, reveal other aspects of macrophage polarization relevant for atherosclerosis treatment.

## 5. Conclusions

The current paradigm suggests that M2 macrophages, owing to their specific localization within human atherosclerotic plaques and their intrinsic anti-inflammatory properties, are primarily associated with plaque stability. By contrast, M1 macrophages are predominant in unstable regions, and their role in atheroinflammation is deleterious. However, the real situation may be more complex, with the identification of several pro- and anti-inflammatory functional states with distinct and, in most cases, not fully identified roles in atherogenesis. A direct causative link between macrophage phenotype and progression of atherosclerotic lesions in humans has not been established, with available information limited to correlation studies. There is a high need for the development of new animal models because the clear proof of the concept of macrophages guiding the evolution and regression of plaques may open up a new area in drug development for the treatment and prevention of atherosclerosis. An improved definition of specific macrophage phenotypes and the role they play in atherogenesis may also be useful for the development of new therapeutic modalities. Due to the high plasticity of macrophages and overlap of macrophage subtype markers, the most perspective strategy seems to be not the targeting or elimination of distinct subpopulations, but modulation of polarizing signals and differentiation pathways, as well as enhancing specific atheroprotective activities.

## Figures and Tables

**Table 1:** Macrophage subtypes and their known functions in atherosclerosis.

Macrophage Subtype	Differentiation Stimuli	Markers (Human)	Markers (Murine)	Functional Activity in Atherosclerosis
M1	Th1 cytokines, cholesterol crystals, lipopolysaccharide, oxLDL, hypoxia, AGE, iron overload	IL-1β, TNF, IL-6, IL-12, IL-23, CXCL9, CXCL10, CXCL11	IL-1β, TNF, IL-6, IL-12, IL-23, CXCL9, CXCL10, CXCL11, arginase II	Pro-inflammatory; high ROS production; recruitment of Th1 cells; necrotic core formation; decreased plaque stability
M2a	Th2 cytokines (IL-4, IL-13)	MMR, IL-1Ra, factor XIIIa, CD200R, CCL18, stabilin-1, CD163	Arginase I, resistin-like α, Ym1, Ym2, MMGL, MMR, stabilin-1, CD163, dectin-1	Resistant to lipid accumulation; anti-inflammatory; tissue repair; pro-fibrotic
M2b	Immune complexes and IL-1β or lipopolysaccharide	IL-10^high^, IL-12^low^	IL-10^high^, IL-12^low^	Resistant to lipid accumulation; anti-inflammatory
M2c	IL-1, TGF-β, glucocorticoids	MMR, MerTK	Arginase I	Resistant to lipid accumulation; effective efferocytosis; anti-inflammatory
M2d	Adenosine A2 receptor agonists, TLR agonists	Not identified	IL-10, iNOS, VEGF	Pro-angoigenic
M(Hb)	Haemoglobin-haptoglobin complexes	CD163, MMR	CD163, MMR	Resistant to lipid accumulation; pro-angoigenic
Mhem	Haem	CD163, ATF-1	CD163, ATF-1	Resistant to lipid accumulation; pro-angoigenic
HA-mac	Haemoglobin-haptoglobin complexes	CD163, HLA-DR^low^	CD163, HLA-DR^low^	Pro-angiogenic
Mox	Oxidized phospholipids			Antioxidant; reduced phagocytic activity
M4	CXCL4	MMP-7, S100-A8, MMR	IL-6, TNF, MMP-7, S100-A8, MMR	Low phagocytic activity; pro-inflammatory; resistant to foam cell formation

AGEs, Advanced glycation end products; ATF-1, cyclic AMP-dependent transcription factor-1; CCL18, C-C motif chemokine ligand 18; CXCL, C-X-C motif chemokine ligand; HLA, human leukocyte antigen; HO 1, haem oxygenase 1; IL, interleukin; iNOS, inducible nitric oxide synthase; MerTK, Mer tyrosine kinase; MMGL, C type lectin domain family 10 member A (also known as MGL 1); MMP 7, matrix metalloproteinase 7; MMR, macrophage mannose receptor; NFE2L2, nuclear factor (erythroid-derived 2)-like 2; TGF β, transforming growth factor β; TH1, type 1 T helper cells; TH2, type 2 T helper cells; TLR, toll-like receptor; TNF, tumor necrosis factor; TR, thioredoxin reductase 1, cytoplasmic; VEGF, vascular endothelial growth factor.

**Table 2:** Classification of murine intraplaque macrophages according to single-cell analysis.

Type	Resident-Like Macrophages	Inflammatory Macrophages	TREM2hi Macrophages
Markers	Lyve1, Cxcr1, Folr2, Cd206, F13a1, Cbr2, Sepp1, Cxcl4, Gas6, Mafb	Tnf, Nlrp3, Il1b, Egr1, Cepbp, Cxcl1, Ccl2–5, Nfkbia	Trem2, Cd9, Lgals3, Ctsb, Spp1
Functional pathways	Endocytosis, proliferation, anti-inflammatory	Inflammatory response	Cholesterol metabolism, oxidative phosphorylation, lipid accumulation, anti-inflammatory
Localization	Adventitia	Intima, plaque shoulder	Intima, necrotic core
Markers of corresponding population in human atherosclerosis	Cd206, Cd163	Hla-dra, Cd74, Cyba, Lyz2, Aif1, S100A8/A9, Malat1, JunB, Nfkbia	Apoc1, ApoE, Ctsb, Fabp5, Plin2, Lgals3, Trem2, Cd9, Lxr, Stat6

Aif, Apoptosis-Inducing Factor; ApoE, Apoliprotein E; Apoc1, apolipoprotein C1; Cbr2, Carbonyl reductase; Ccl, C-C motif ligand; Cepbp, CCAAT/enhancer-binding protein beta; Ctsb, Cathepsin B; Cxcl4, C-X-C motif ligand 4; Cx3cr1, C-X3-C Motif Chemokine Receptor 1; Cyba, Cytochrome B-245 Alpha Chain; Egr1, Early Growth Response 1; F13a1, factor XIIIa; Fabp5, Fatty Acid Binding Protein 5; Folr2, Folate Receptor Beta; Gas6, Growth Arrest Specific 6; IL1b, Interleukin 1 beta; Lgals3, Galectin-3; Lxr, Liver X receptor; Lyve1, lymphatic vessel endothelial hyaluronan receptor 1; Lyz2, Mafb, MAF BZIP Transcription Factor B; Malat1, Metastasis Associated Lung Adenocarcinoma Transcript 1; Nfkbia, NFKB Inhibitor Alpha; Nlrp3, NLR Family Pyrin Domain Containing 3; Plin2, Perilipin 2; Sepp1, Selenoprotein P; Spp1, Secreted Phosphoprotein 1; S100A8, S100 Calcium Binding Protein A8; Stat6, Signal Transducer And Activator Of Transcription 6; Tnf, Tumor Necrosis Factor, Trem2, Triggering Receptor Expressed On Myeloid Cells 2.

## Data Availability

Not applicable.
